# Fcγ-receptor IIa-mediated Src Signaling Pathway Is Essential for the Antibody-Dependent Enhancement of Ebola Virus Infection

**DOI:** 10.1371/journal.ppat.1006139

**Published:** 2016-12-30

**Authors:** Wakako Furuyama, Andrea Marzi, Aaron B. Carmody, Junki Maruyama, Makoto Kuroda, Hiroko Miyamoto, Asuka Nanbo, Rashid Manzoor, Reiko Yoshida, Manabu Igarashi, Heinz Feldmann, Ayato Takada

**Affiliations:** 1 Division of Global Epidemiology, Research Center for Zoonosis Control, Hokkaido University, Sapporo, Japan; 2 Laboratory of Virology, Division of Intramural Research, National Institute of Allergy and Infectious Diseases, National Institutes of Health, Rocky Mountain Laboratories, Hamilton, MT, United States of America; 3 Research Technologies Branch, Division of Intramural Research, National Institute of Allergy and Infectious Diseases, National Institutes of Health, Hamilton, MT, United States of America; 4 Department of Cell Physiology, Hokkaido University Graduate School of Medicine, Sapporo, Japan; 5 Global Station for Zoonosis Control, Global Institution for Collaborative Research and Education, Hokkaido University, Sapporo, Japan; 6 School of Veterinary Medicine, the University of Zambia, Lusaka, Zambia; University of Texas Medical Branch, UNITED STATES

## Abstract

Antibody-dependent enhancement (ADE) of Ebola virus (EBOV) infection has been demonstrated in vitro, raising concerns about the detrimental potential of some anti-EBOV antibodies. ADE has been described for many viruses and mostly depends on the cross-linking of virus-antibody complexes to cell surface Fc receptors, leading to enhanced infection. However, little is known about the molecular mechanisms underlying this phenomenon. Here we show that Fcγ-receptor IIa (FcγRIIa)-mediated intracellular signaling through Src family protein tyrosine kinases (PTKs) is required for ADE of EBOV infection. We found that deletion of the FcγRIIa cytoplasmic tail abolished EBOV ADE due to decreased virus uptake into cellular endosomes. Furthermore, EBOV ADE, but not non-ADE infection, was significantly reduced by inhibition of the Src family protein PTK pathway, which was also found to be important to promote phagocytosis/macropinocytosis for viral uptake into endosomes. We further confirmed a significant increase of the Src phosphorylation mediated by ADE. These data suggest that antibody-EBOV complexes bound to the cell surface FcγRIIa activate the Src signaling pathway that leads to enhanced viral entry into cells, providing a novel perspective for the general understanding of ADE of virus infection.

## Author Summary

Antibody-dependent enhancement (ADE), a phenomenon in which viral infectivity is increased by virus-specific antibodies, is observed in vitro for a large number of viruses. For some of these viruses, ADE often become an issue for disease control by vaccination. It has also been shown that some human sera convalescent from Ebola virus disease contain ADE antibodies. ADE has been shown mostly to depend on the cross-linking of virus-antibody complexes to cell surface Fc receptor, which activate various signaling pathways involved in the reorganization of the actin cytoskeleton and membrane remodeling. In this study, we demonstrate that Fc receptor-mediated intracellular signaling is a key factor for ADE of Ebola virus infection. We found that the antibody-virus complexes bound to the cell surface Fc receptors triggered the phosphorylation of particular protein-tyrosine kinases that activated signaling pathways leading to enhanced viral uptake into cells through phagocytosis and/or macropinocytosis. Our study provides new insights into mechanisms of ADE and also offer a potential new cellular target to develop treatments for ADE-associated diseases such as dengue hemorrhagic fever and possibly Zika virus infection.

## Introduction

Ebola virus (EBOV), a member of the family *Filoviridae*, causes severe hemorrhagic fever in humans and nonhuman primates, with human case fatality rates of up to 90% [1]. EBOV expresses a glycoprotein (GP) that is the only viral surface protein and important for both receptor binding and membrane fusion [2,3]. EBOV entry is initiated by viral attachment to cell surface molecules such as T-cell immunoglobulin and mucin domain 1 (TIM-1) and C-type lectins [4,5], followed by internalization of the virus particle into cells via macropinocytosis [6–8]. In the late endosome, EBOV GP is cleaved by host proteases such as cathepsins L and B [9], exposing the GP receptor binding site that then binds to the receptor, Niemann-Pick C1 (NPC1), followed by membrane fusion [10,11].

In addition to the direct interaction between GP and host cell receptors, it has been demonstrated that EBOV exploits some GP-specific antibodies for its entry into cells, leading to increased infectivity *in vitro* [12,13]. This phenomenon has been described for a number of viruses and is known as antibody-dependent enhancement (ADE) [14–17]. For some of these viruses, ADE has become a great concern to disease control by vaccination. Particularly, convalescent human sera have been shown to contain ADE antibodies [12,13], raising concerns about potential detrimental effects of passive immunization with convalescent human sera, which is currently under consideration for treatment of Ebola virus disease. Importantly, it was recently demonstrated that therapeutic treatment with convalescent sera having in vitro neutralizing activities was not sufficient for protection against EBOV infection in nonhuman primates [18]. Although ADE was not evaluated in vitro and any enhanced pathogenicity in the treated animals was not observed, it might be possible that ADE antibodies counterbalanced the neutralizing activity as suggested previously [17]. Two distinct pathways of EBOV ADE, one mediated by Fc receptors and the other by complement component C1q and its ligands, are known [13,17]. In particular, the Fcγ receptor (FcγR) is commonly involved in ADE of virus infections [19,20]. However, the molecular mechanisms underlying ADE-mediated virus entry through FcγR are not fully understood.

Three classes of FcγR, FcγRI (CD64), FcγRII (CD32), and FcγRIII (CD16), are expressed in various human immune cells such as dendritic cells, monocytes, and B lymphocytes [21]. Among these FcγRs, FcγRII is a key molecule for EBOV ADE of infection in human leukemia K562 cells [17]. Human FcγRII exists in two isoforms, FcγRIIa and FcγRIIb, which differ in their signal peptides and cytoplasmic tails. FcγRIIa is the active form of FcγRII and contains an immunoreceptor tyrosine-based activation motif (ITAM) in its cytoplasmic tail [21]. The cytoplasmic tail of FcγRIIa is known to contribute to the activitation of two structurally and functionally distinct protein-tyrosine kinase (PTK) classes, the sarcoma (Src) family PTKs [22,23] and spleen tyrosine kinase (Syk) [24]. In addition, Syk is reported to participate in activation of enzymes such as rat sarcoma (Ras), phosphatidylinositol 3-kinase (PI3K), and Bruton’s tyrosine kinase (Btk) [21,25]. These signaling pathways are known to be important for the induction of phagocytic and endocytic processes to internalize immune complexes [21,25,26].

In this study, we focused on the role of FcγRIIa and investigated the contribution of FcγRIIa-mediated signaling to the ADE of EBOV infection. We show that Src family PTKs are essential for EBOV ADE-mediated entry. Our data indicate that binding of antibody-virus complexes to the cell surface FcγRIIa triggers phosphorylation of Src family PTKs and activates subsequent signaling pathways, leading to enhanced viral uptake through phagocytosis and/or macropinocytosis.

## Results

### Cytoplasmic tail of FcγRIIa is required for ADE of EBOV infection

**Fig 1:**
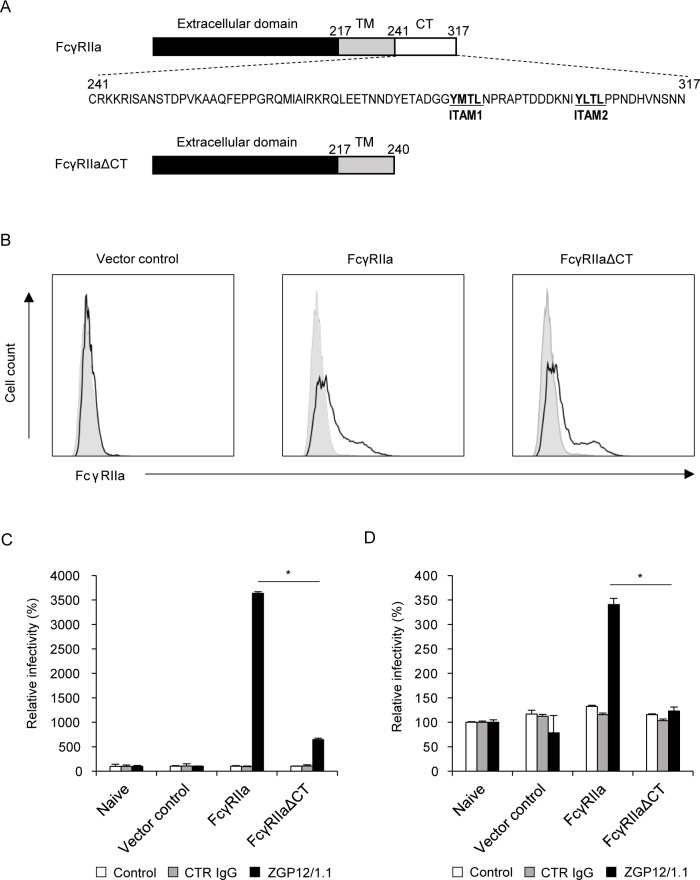
Importance of FcγRIIa and its cytoplasmic tail in EBOV ADE. (A) Schematic representation of FcγRIIa and FcγRIIaΔCT. TM, transmembrane region; CT, cytoplasmic tail. (B) Cell surface expression of the FcγRIIa ectodomain on Jurkat T cell lines. Jurkat T cells stably expressing FcγRIIa and FcγRIIaΔCT were stained with an anti-CD32 antibody and analyzed by flow cytometry. Black lines represent vector-transduced, FcγRIIa-, and FcγRIIaΔCT-expressing cells. Gray shading represents naive Jurkat T cells. (C, D) Infectivity of VSV-EBOV GP and EBOV in Jurkat T cell lines. VSV-EBOV GP (C) and EBOV (D) were incubated with medium alone (Control), CTR IgG, or ZGP12/1.1, followed by inoculation into each Jurkat T cell line. At 24 h (C) and 72 h (D) after inoculation, GFP-positive cells were counted. The relative infectivity in each cell line was calculated by setting the IU value of untreated (Control), CTR IgG-, and ZGP12/1.1-treated viruses in naive Jurkat T cells to 100%, respectively. The mean and standard deviation of three independent experiments are shown. Statistical analysis was performed using Student’s *t*-test (*p<0.05).

To investigate the role of FcγRIIa and, in particular, the importance of its cytoplasmic tail in EBOV ADE, we compared the functions of wild-type FcγRIIa and a deletion mutant of FcγRIIa lacking its cytoplasmic tail (FcγRIIaΔCT). Both molecules were expressed on Jurkat T cells, which are known to be poorly permissive for EBOV infection [27] and lack this Fc receptor [28]. Jurkat T cells were transduced with full-length FcγRIIa or FcγRIIaΔCT genes using a retrovirus vector (Fig 1A and 1B) and subsequently infected with vesicular stomatitis virus (VSV) pseudotyped with EBOV GP (VSV-EBOV GP) and infectious EBOV in the presence or absence of the GP-specific monoclonal antibody (MAb) ZGP12/1.1, which is known to induce EBOV ADE [12] (Fig 1C and 1D). We found that viral infectivity was almost undetectable in naive and control vector-transduced Jurkat T cells but the expression of wild-type FcγRIIa significantly enhanced the infectivity of VSV-EBOV GP and EBOV in the presence of ZGP12/1.1, though not control IgG (CTR IgG). Interestingly, the infection rate of FcγRIIaΔCT-expressing cells was significantly lower than that of cells expressing wild-type FcγRIIa. These results indicated that the FcγRIIa-MAb complex functioned as a receptor-like molecule on this poorly permissive cell line and efficiently promoted infection through the ADE of EBOV entry. More importantly, the results suggested that signaling pathways via the FcγRIIa cytoplasmic tail were likely involved in the ADE of EBOV entry into cells.

### Cytoplasmic tail of FcγRIIa is important for enhanced viral uptake

**Fig 2:**
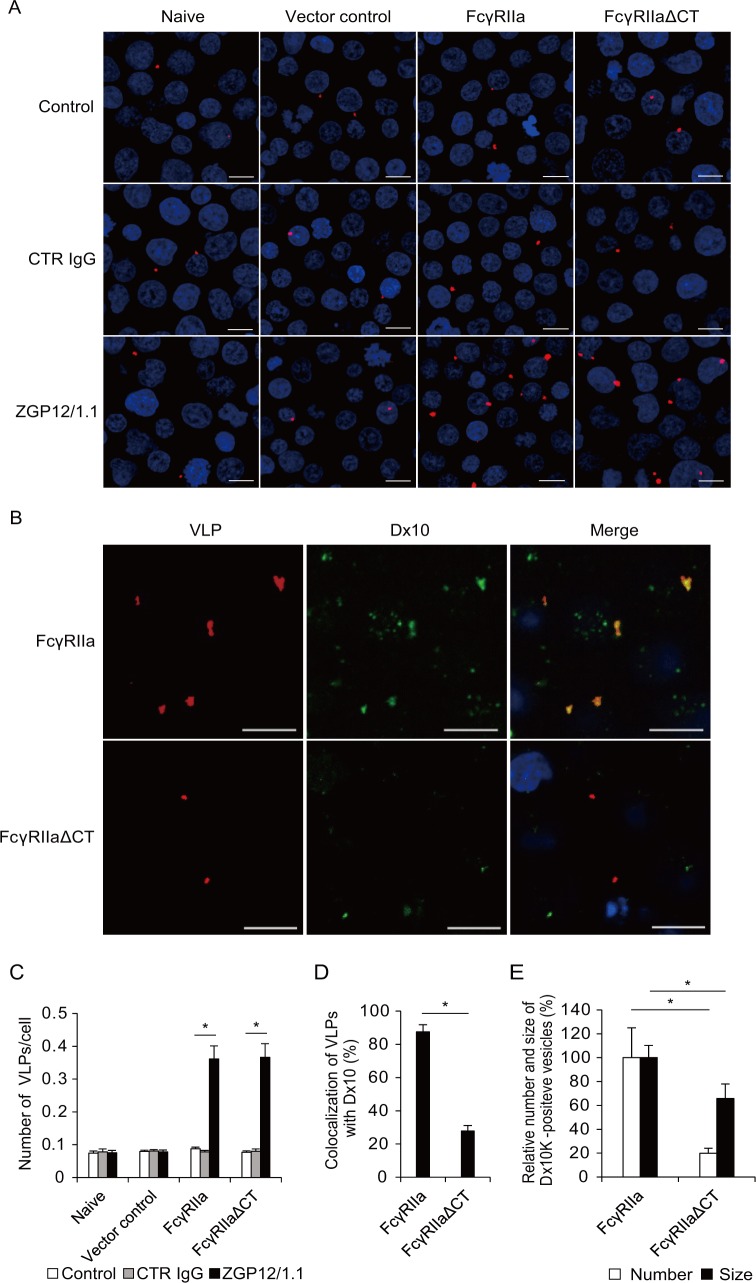
Importance of FcγRIIa and its cytoplasmic tail in ADE-mediated VLP uptake into cells. (A, B) Fluorescent images of attachment and internalization of VLPs. (C-E) Quantified fluorescent signals of VLP and Dx10. Untreated (Control), CTR IgG-, and ZGP12/1.1-treated DiI-labeled VLPs were inoculated into each Jurkat T cell line and incubated for 30 min on ice. After adsorption, the cells were incubated for 0 h (A, C) or incubated with Alexa647-labeled Dx10 for 2 h at 37°C (B, D, E). VLPs (red) on the cell surface (A, C) and VLPs (red) and Dx10 (green) in the cytoplasm (B, D, E) were monitored by confocal laser scanning microscopy. Scale bars represent 10 μm. Nuclei of cells are visualized with DAPI (blue). The number of VLPs attached to the cell surface (C), the colocalization of VLPs (DiI) and Dx10 (Alexa647) signals (D), and the number and size of Dx10-positive vesicles (E) were quantified. The number and size were calculated by setting the value of FcγRIIa-expressing Jurkat T cells in the presence of ZGP12/1.1-treated VLPs to 100%. The mean and standard deviation of three independent experiments are shown. Statistical analysis was performed using Student’s *t*-test (*p<0.05).

FcγRIIa is known to modulate phagocytosis/macropinocytosis through signaling pathways via its cytoplasmic tail [29,30]. Therefore, to analyze viral binding and intracellular uptake in more detail, we produced lipophilic tracer (DiI)-labeled virus-like particles (VLPs) consisting of the major EBOV structural proteins, GP, matrix protein (VP40), and nucleoprotein (NP), and monitored the localization of VLPs in each transduced Jurkat T cell line (Fig 2). The number of VLPs attached to the surface of naive and empty vector-transduced Jurkat T cells was not significantly different irrespective of the presence of CTR IgG and ZGP12/1.1 (Fig 2A and 2C). In contrast, the attachment of VLPs was significantly enhanced to similar extents in Jurkat T cells expressing FcγRIIa and FcγRIIaΔCT in the presence of ZGP12/1.1 but not CTR IgG (Fig 2A and 2C), suggesting that the FcγRIIa ectodomain expressed on Jurkat T cells had the ability to increase the VLP attachment mediated by ZGP12/1.1. Next, we assessed the number of VLPs incorporated into intracellular vesicles along with Alexa Fluor 647 (Alexa647)-labeled dextran Mw 10,000 (Dx10), a specific probe for visualizing phagocytotic and macropinocytotic vesicles [6,31]. After incubation for 2 h, ZGP12/1.1-treated VLPs efficiently colocalized with Dx10 in Jurkat T cells expressing wild-type FcγRIIa, but not in cells expressing FcγRIIaΔCT (Fig 2B and 2D). Viral uptake was not observed drastically in the absence of FcγRIIa and ZGP12/1.1. Furthermore, the number and size of Dx10-positive vesicles incorporated into Jurkat T cells expressing FcγRIIaΔCT were significantly smaller than those in cells expressing FcγRIIa, indicating the importance of the FcγRIIa cytoplasmic tail in activating the phagocytosis/macropinocytosis (Fig 2B and 2E). These data suggested that the ADE infection of FcγRIIa-expressing Jurkat T cells was associated with enhanced viral uptake into cellular vesicles, most likely due to the activation of FcγRIIa-mediated signaling via its cytoplasmic tail.

### Intracellular signaling via Src family PTKs contributes to ADE of EBOV infection

**Fig 3:**
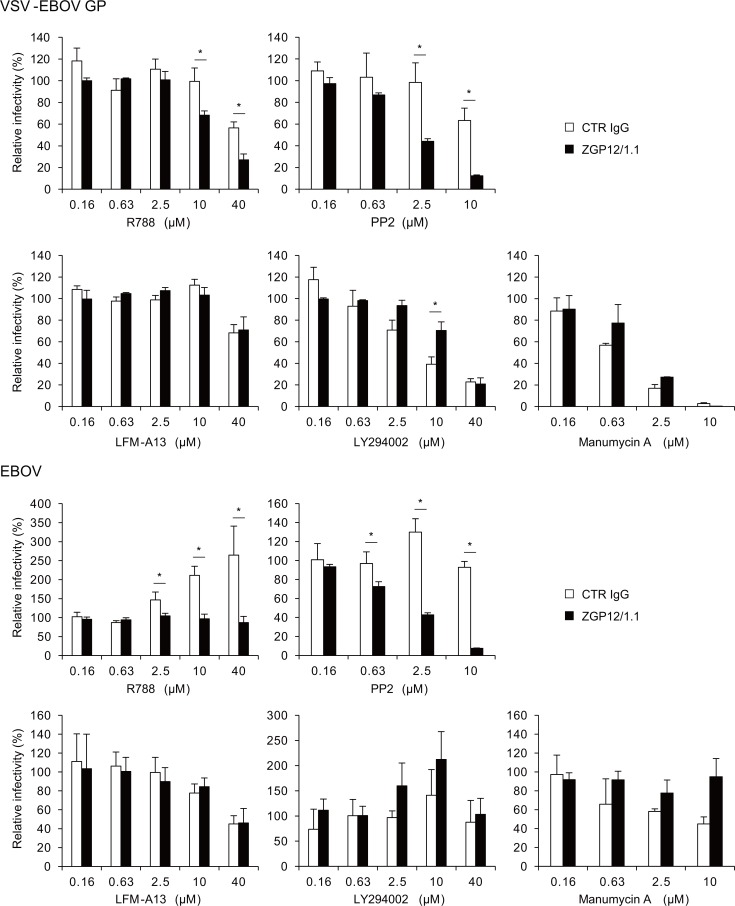
Identification of FcγRIIa-mediated signaling pathway for EBOV ADE. K562 cells were treated with the indicated concentrations of R788, PP2, LFM-A13, LY294002, or Manumycin A for 1 h at 37°C and inoculated with VSV-EBOV GP or EBOV preincubated with CTR IgG, or ZGP12/1.1. The relative percentage of infectivity was calculated by setting the IU value of the viruses in DMSO or ethanol-treated cells to 100%. The mean and standard deviation of three independent experiments are shown. Statistical analysis was performed using Student’s *t*-test (*p<0.05).

To identify the intracellular signaling pathway involved in the ADE of EBOV entry, we analyzed the effects of different inhibitors of signaling pathways in K562 cells, which naturally express FcγRIIa. Consistent with previous studies [17,32], K562 cells were permissive to VSV-EBOV GP and EBOV infections, and viral infection rates were significantly enhanced in the presence of ZGP12/1.1 (S1A and S1B Fig). We then tested inhibitors of Syk and Src family PTKs (R788 and PP2, respectively) as these PTKs are known to be principally involved in signaling pathways downstream of FcγRIIa, and in particular to play important roles in inducing FcγR-mediated phagocytosis/macropinocytosis [26,33]. We found that the ADE of VSV-EBOV GP infection was significantly reduced in K562 cells treated with these inhibitors in a dose-dependent manner. In contrast, only a limited reduction was seen in non-ADE infection at the highest concentrations of the inhibitors (Fig 3). The ADE-specific inhibitory effect was more prominent in cells treated with PP2 than in those treated with R788.

Since Syk is reported to participate in the activation of signaling through PI3K, Btk, and Ras, we further examined which pathways downstream of Syk contributed to the ADE of VSV-EBOV GP infection using specific inhibitors of PI3K (LY294002), Btk (LFM-A13), and Ras (Manumycin A) (Fig 3). However, both ADE and non-ADE infections by VSV-EBOV GP were dose-dependently reduced by LY294002 and Manumycin A, respectively. LFM-A13 showed little effect on the infectivity of VSV-EBOV GP. Subsequently, we confirmed the effects of these inhibitors using infectious EBOV. Consistent with the data for VSV-EBOV GP, PP2 selectively reduced the ADE, but not the non-ADE infection. Interestingly, R788 showed no effects on the ADE of EBOV infection and rather enhanced the non-ADE infection. LFM-A13 slightly reduced both the ADE and the non-ADE infections, whereas LY294002 and Manumycin A did not inhibit the ADE infection, though Manumycin A slightly inhibited the non-ADE infection.

**Fig 4:**
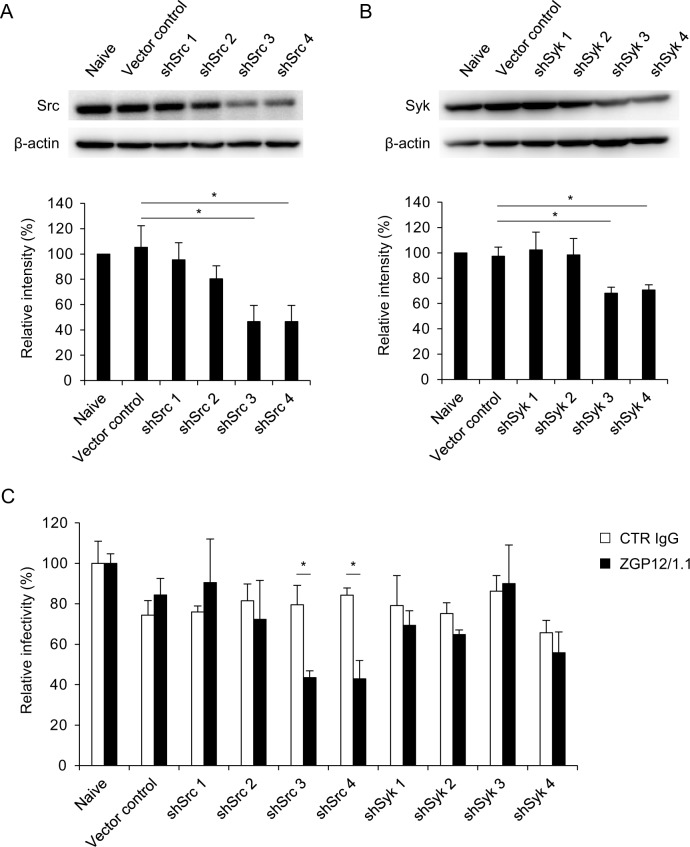
Effects of Src and Syk knockdown on EBOV ADE. (A, B) Validation of knockdown efficiency of Src and Syk in K562 cell lines. (C) Relative infectivity of VSV-EBOV GP in K562 cell lines. Src (A) and Syk (B) proteins in each cell line were detected by Western blotting. The band intensity of Src and Syk was standardized with that of the β-actin band for each cell line. Relative intensities of the Src and Syk bands were then calculated by setting the intensity of naive K562 cells to 100%. These cell lines were infected with VSV-EBOV GP preincubated with CTR IgG or ZGP12/1.1 (C). The relative percentage of infectivity was calculated by setting the IU value of the viruses in naïve K562 cells to 100%. The mean and standard deviation of three independent experiments are shown. Statistical analysis was performed using Student’s *t*-test (*p<0.05).

To further investigate the role of Src- and Syk-mediated signaling in EBOV ADE, we generated Src and Syk knockdown K562 cells. K562 cells were transduced with retroviral vectors expressing small hairpin RNAs (shRNAs) for silencing Src or Syk genes (Fig 4A and 4B) and infected with VSV-EBOV GP in the presence or absence of ZGP12/1.1 (Fig 4C). We found that transduced cells stably expressing Src shRNAs (shSrc3 and shSrc4) showed approximately 50% reduction in protein levels (Fig 4A) and ZGP12/1.1-mediated ADE was significantly decreased in these cell lines (Fig 4C). In contrast, no significant difference was seen between ADE (ZGP12/1.1) and non-ADE (CTR IgG) infections in Syk knockdown cells although 2 of the Syk shRNAs (shSyk3 and shSyk4) significantly reduced the expression of the Syk protein (Fig 4B and 4C).

These results demonstrated that FcγRIIa-mediated signaling through the activation of Src family PTKs contributed to the ADE of EBOV infection, but Syk-related signaling including PI3K, Btk, and Ras did not seem to be specifically involved. We further tested the effect of PP2 in K562 cell lines stably expressing dendritic cell-specific ICAM-3-grabbing non-integrin (DC-SIGN) or human macrophage galactose-type C-type lectin (hMGL), both of which have been shown to act as attachment receptors for EBOV [32, 34], and found that PP2 had limited effects on the infectivity of VSV-EBOV GP in these cell lines (S2 Fig).

### VLP-MAb complexes activate phosphorylation of Src in K562 cells

**Fig 5:**
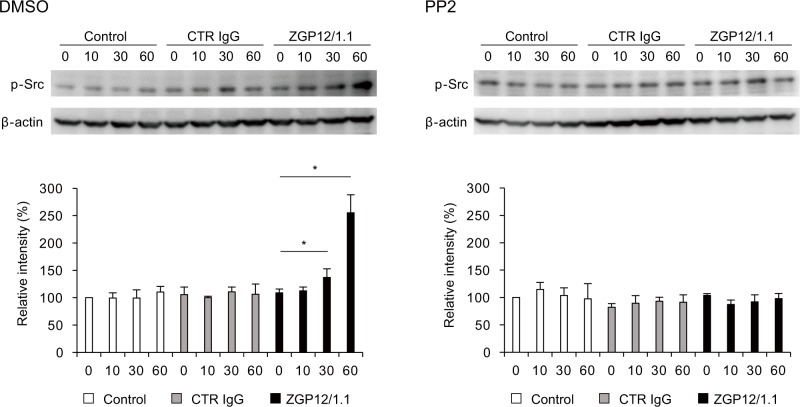
Increased phosphorylation of Src in K562 cells exposed to the VLP-ZGP12/1.1 complex. Purified VLPs were preincubated with medium alone (Control), CTR IgG, or ZGP12/1.1 and inoculated into K562 cells pretreated with DMSO or PP2 for 1 h at 37°C. At 0, 10, 30, and 60 min postinoculation, phosphorylation of Src at Tyr416 (p-Src) was detected by Western blotting. The band intensity of p-Src was standardized with that of the β-actin band at each time point. Relative intensities of the p-Src bands were then calculated by setting the intensity of control cells (0 h) to 100%. The mean and standard deviation of three independent experiments are shown. Statistical analysis was performed using Student’s *t*-test (*p<0.05).

To directly detect the activation of Src family PTKs, we quantified the phosphorylation levels of Src in K562 cells (Fig 5). We found no significant difference in the cells inoculated with intact VLPs alone at each time point. Likewise, inoculation of CTR IgG-treated VLPs did not enhance Src phosphorylation levels. However, a significant increase of the Src phosphorylation was detected at 30 and 60 min after K562 cells were exposed to ZGP12/1.1-treated VLPs (Fig 5 left). Furthermore, the enhanced phosphorylation was completely blocked by the Src family PTK inhibitor, PP2 (Fig 5 right). These findings suggested that Src were activated by the interaction of the VLP-ZGP12/1.1 complex with FcγRIIa.

### The ADE of EBOV entry depends on phagocytosis and/or macropinocytosis mediated by Src family PTKs

**Fig 6:**
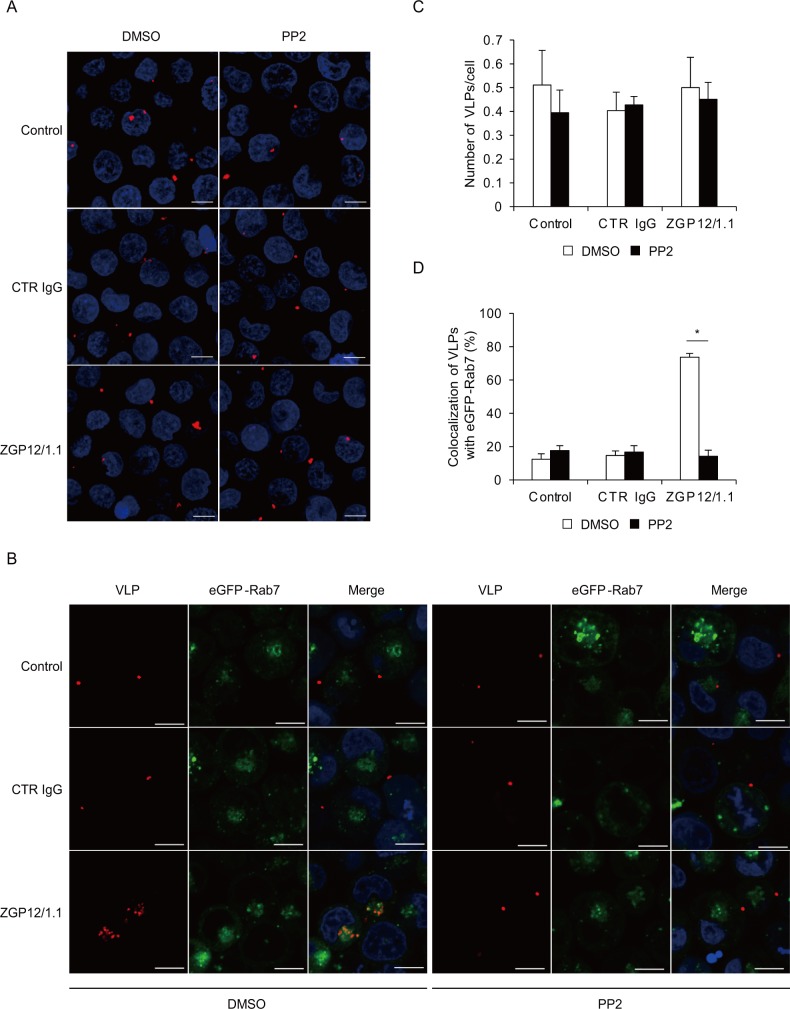
Enhanced VLP uptake into endosomes in K562 cells during ADE. (A, B) Fluorescent images of attachment and internalization of VLPs. (C, D) Quantified fluorescent signals of VLP and Rab-7. K562 cells expressing eGFP-Rab7 were incubated with DMSO or PP2 for 1 h at 37°C. Untreated (Control), CTR IgG-, and ZGP12/1.1-treated DiI-labeled VLPs were inoculated into cells and VLPs (red) on the cell surface at 0 h (A, C) and VLPs (red) and eGFP-Rab7 (green) in the cytoplasm at 2 h (B, D) after adsorption were monitored by confocal laser scanning microscopy. Scale bars represent 10μm. Nuclei of cells are visualized with DAPI (blue). The number of VLPs on the cell surface (C) and the colocalization of VLPs (DiI) and eGFP-Rab7 signals (D) were quantified. The mean and standard deviation of three independent experiments are shown. Statistical analysis was performed using Student’s *t*-test (*p<0.05).

To further characterize the role of the Src family PTK-dependent signaling in the ADE of EBOV infection, we analyzed the effect of PP2 on the attachment and uptake of DiI-labeled VLPs using K562 cells. We first compared the number of VLPs attached to the cell surface among untreated, CTR IgG-, and ZGP12/1.1-treated VLPs. Since the overexpression of FcγRIIa in Jurkat T cells increased the attachment of VLPs to the cell surface in the presence of ZGP12/1.1 (Fig 2), we hypothesized that ZGP12/1.1 would enhance the VLP attachment to K562 cells. However, the number of VLPs attached to the cell surface was not significantly different in the presence or absence of ZGP12/1.1 (Fig 6A left and 6C), and was not affected by the PP2 treatment (Fig 6A right and 6C), indicating that EBOV ADE in K562 cells did not result from increased viral attachment to the cell surface. For the visualization of the VLP uptake into endosomes, K562 cells expressing enhanced green fluorescent protein fused to Rab7 (eGFP-Rab7), a late endosome marker, were used to analyze colocalization of eGFP-Rab7 and internalized VLPs. We found that ZGP12/1.1-treated VLPs were efficiently colocalized with eGFP-Rab7 in K562 cells, whereas only 10–20% colocalization was seen in the cells inoculated with untreated or CTR IgG-treated VLPs (Fig 6B left, 6D and S3A Fig). We further found that the enhanced colocalization of eGFP-Rab7 and ZGP12/1.1-treated VLPs was clearly blocked by the PP2 treatment (Fig 6B right, 6D and S3B Fig). These results indicated that the ADE of EBOV entry into K562 cells was dependent on the Src family PTK activation leading to increased uptake of viral particles into cells.

**Fig 7:**
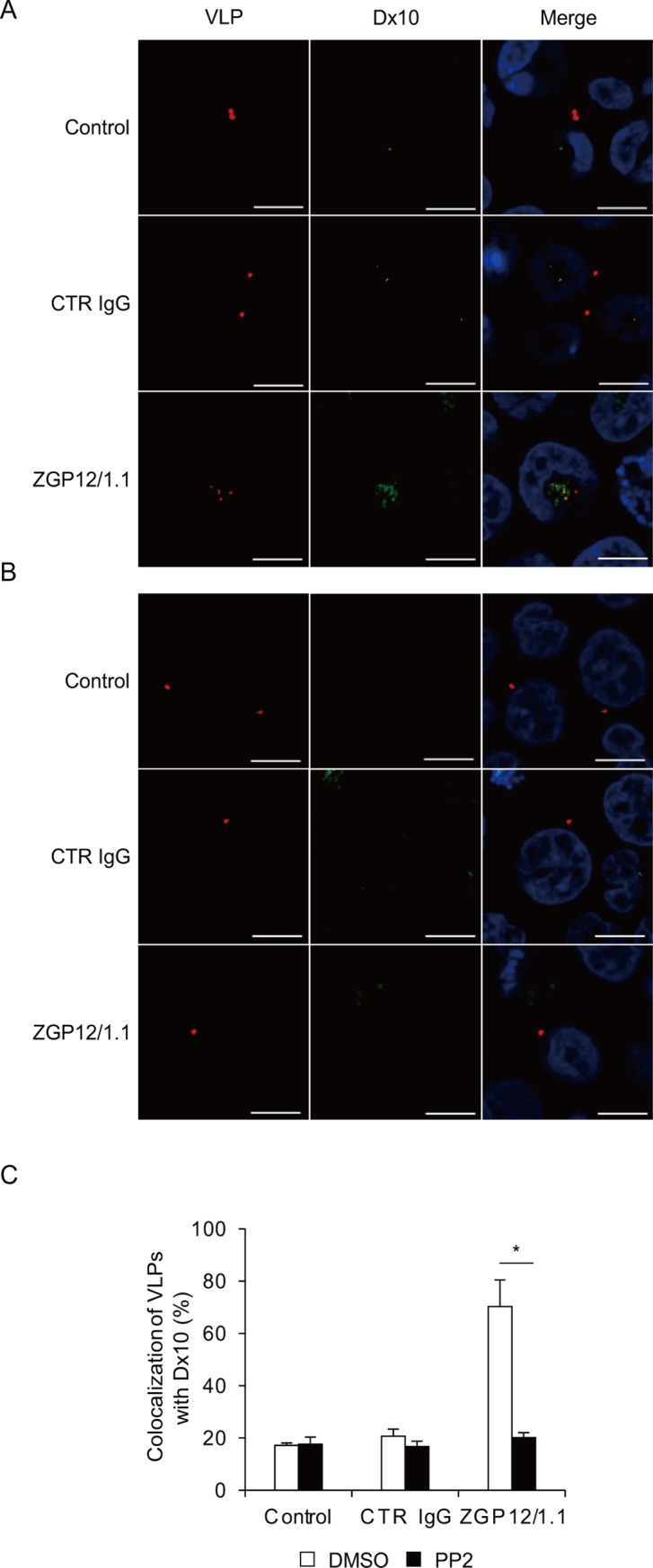
Colocalization of Dx10 and ZGP12/1.1-treated VLPs. (A, B) Fluorescent images of internalization of VLPs and Dx10. c, Quantified fluorescent signals of VLPs and Dx10. K562 cells were incubated with DMSO (A) or PP2 (B) for 1 h at 37°C. Untreated (Control), CTR IgG-, and ZGP12/1.1-treated DiI-labeled VLPs were inoculated into cells and incubated for 30 min on ice. After adsorption, cells were incubated with Alexa647-labeld Dx10 for 1 h at 37°C in the presence of DMSO (A) or PP2 (B). VLPs (red) and Dx10 (green) in the cytoplasm were monitored by confocal laser scanning microscopy. The colocalization of VLPs (DiI) and Dx10 (Alexa647) signals was quantified (C). Scale bars represent 10 μm. Nuclei of cells are visualized with DAPI (blue). The mean and standard deviation of three independent experiments are shown. Statistical analysis was performed using Student’s *t*-test (*p<0.05).

Finally, we investigated whether the ADE of EBOV entry into K562 cells depended on phagocytosis/macropinocytosis, which have been shown to be major pathways of the EBOV entry through non-ADE pathways [6–8]. We used Dx10 to visualize phagocytotic and macropinocytotic vesicles in K562 cells and analyzed its colocalization with VLPs. We found that approximately 70% of the ZGP12/1.1-treated VLP signals were overlapped with Dx10 in intracellular vesicles (Fig 7A and 7C and S4A Fig). Furthermore, the colocalization of Dx10 and ZGP12/1.1-treated VLPs was significantly blocked by the PP2 treatment (Fig 7B and 7C and S4B Fig). To confirm that these observations were EBOV-specific, we further analyzed DiI-labeled SUDV VLPs and found that ZGP12/1.1 affected neither attachment/uptake of SUDV VLPs nor Dx10 uptake. (S5 Fig). These results suggested that the surface-bound VLP-ZGP12/1.1 complex was incorporated into cells through Src family PTK-dependent phagocytosis and/or macropinocytosis during the ADE of EBOV entry.

## Discussion

It is well established that after the attachment of virus particles to cell surface receptors a variety of signaling pathways are activated through tyrosine and phosphoinositol kinases, and the subsequent cellular events such as endocytosis, including macropinocytosis and phagocytosis, are important for the entry of viruses [35,36]. Likewise, it has been suggested that signaling pathways via FcγR are involved in ADE of virus infections [20]. However, there is limited information on the detailed molecular mechanisms of intracellular signaling pathways required for ADE of virus infection. It has been shown that the non-ADE entry of EBOV requires host factors such as PI3K, the Rho family, and protein kinase C [6,7,37], although the virus-specific receptor molecules involved in these signaling pathways are not yet identified. In the present study, we focused on the ADE of EBOV entry and found that the FcγRIIa-mediated signaling pathway was essential for this process, which is distinct from those required for the non-ADE entry.

The FcγRIIa cytoplasmic tail has been shown to be essential for ADE of dengue virus entry and the involvement of Syk cascade in ADE entry has been reported [38–40]. Our data indicate that the cytoplasmic tail of FcγRIIa is crucial for the ADE of EBOV infection and that Src family PTK-dependent signaling is important to enhance viral uptake into cellular vesicles during ADE-mediated entry of EBOV. Src family PTKs are non-receptor tyrosine kinases involved in the regulation of diverse cellular functions like proliferation, differentiation, adhesion, and phagocytosis [41,42]. Importantly, this signaling pathway is known to regulate endocytic machinery by triggering the reorganization of the actin cytoskeleton and membrane remodeling [21,26]. Indeed, previous studies have demonstrated that Src family PTK-dependent signaling is required for the non-ADE entry of some viruses into host cells [43,44]. Our data indicate that this signaling is also used to promote viral particle uptake during the FcγRIIa-mediated ADE of EBOV entry.

It was noted that there was a significant increase (approximately 400%) in infectivity of VSV-EBOV GP when FcγRIIaΔCT is expressed on Jurkat T cells, although it was not as high as wildtype FcγRIIa. This observation suggest that the binding provided by the external portion of FcγRIIa may also have some importance and that the signaling function provided by the cytoplasmic portion of FcγRIIa further enhances the ADE effect. It might also be possible that the FcγRIIa associated with lipid rafts activates some FcγRIIa-mediated signals through its transmembrane domain as described previously [45].

Interestingly, we found that the Syk inhibitor R788 reduced the ADE efficiency of VSV-EBOV GP but not EBOV. While the VSV pseudotype system is widely used to study EBOV GP functions, it has been suggested that pseudotyped VSV and authentic EBOV can utilize different entry pathways since the particle size and structure of pseudotyped VSV do not accurately recapitulate those of EBOV [6,46]. Thus, it may be possible that the EBOV entry primarily relies on macropinocytosis as shown previously [6–8], whereas VSV-EBOV GP can also be incorporated into smaller vesicles. We assume that this difference could influence the effect of the inhibitor since Syk-dependent signaling might be associated with caveolin-mediated endocytosis [47,48]. Another difference found between VSV-EBOV GP and authentic EBOV was that while the Syk inhibitor did not affect EBOV ADE, non-ADE infection was significantly enhanced in the presence of this inhibitor, an effect not observed for VSV-EBOV GP. This might be due to the effect on post-entry mechanisms such as antiviral cellular responses, as proposed by a recent study demonstrating that dengue virus-antibody complexes decreased type-I interferon-stimulated gene expression triggered by the FcγR-mediated signaling pathway through Syk, leading to enhanced replication of the virus [39,40]. Since such an antiviral response could be different between VSV- and EBOV-infected cells, it is possible that the Syk inhibitor specifically affected the replication of EBOV, but not VSV, RNA genomes.

Previous studies have demonstrated that cellular C-type lectins such as DC-SIGN and hMGL serve as attachment receptors and promote the entry of EBOV into cells [5,32,34]. These C-type lectins, as well as Fc receptors, are thought to initiate phagocytic pathways for uptake of microorganisms, cell debris, and apoptotic cells [49,50]. Interestingly, both C-type lectins and ADE-antibodies, including ZGP12/1.1, mainly bind to the mucin-like region of EBOV GP, which has a number of N- and O-linked glycosylation sites in the middle portion of the protein [17,32,34], suggesting a similarity in the mechanism of the virus entry mediated by C-type lectins and ADE. However, the Src family PTK inhibitor PP2 showed limited effects on the infectivity of VSV-EBOV GP in the cell lines expressing DC-SIGN or hMGL. Taken together, our data suggest that the FcγRIIa-mediated EBOV ADE principally depends on signaling pathways distinct from those for C-type lectin-mediated entry, while both C-type lectins and ADE-antibody-FcγRIIa complexes are assumed to serve as attachment receptors and subsequent processes for membrane fusion (i.e., cathepsin cleavage and NPC1 binding) appear also to be similar.

In conclusion, our data indicate that EBOV ADE is not simply dependent on increased viral attachment through interaction between FcγRIIa and virus-antibody complexes, and that the induction of FcγRIIa-mediated signaling associated with the activation of Src family PTKs is essential for EBOV ADE. This signaling pathway most likely promotes macropinocytosis, the major entry pathway of EBOV, and leads to enhanced viral uptake into cells. The contribution of Src family PTKs to FcγRIIa-mediated ADE of virus entry has not been demonstrated previously. Discovery of this ADE mechanism provides a novel perspective for the general understanding of ADE of virus infection. Although the impact of ADE on disease progression remains unclear for many viruses, our findings may offer a potential new target to develop treatments for ADE-associated diseases such as dengue hemorrhagic fever and possibly Zika virus infection [16,51–53] since signaling pathways are known to be essential for virus entry into cells and some signaling inhibitors have been considered to be potential treatment options for virus infections [54–56]. However, since non-ADE entry mechanisms of these viruses are different from EBOV, it is required to investigate whether the Src family PTK-mediated ADE mechanism can be generally applied for other viruses known to utilize ADE entry into cells.

## Materials and Methods

### Cells and viruses

African green monkey kidney Vero E6 cells and human embryonic kidney (HEK) 293T cells were grown in Dulbecco’s modified Eagle’s medium (DMEM) (Sigma), and human chronic myelogenous leukemia K562, K562/DC-SIGN, and K562/hMGL cell lines [32,57] and human leukemic Jurkat T cells were grown in Roswell Park Memorial Institute (RPMI) 1640 medium (Sigma). These media were supplemented with 10% fetal calf serum (FCS) (Cell Culture Bioscience), 100 U/ml penicillin, and 0.1 mg/ml streptomycin (Gibco). These cells were obtained from an already-existing collection in the Research Center for Zoonosis Control, Hokkaido University. EBOV expressing GFP [58] was propagated in Vero E6 cells and stored at -80°C. Replication-incompetent VSV pseudotyped with EBOV GP containing GFP instead of the VSV G gene (VSV-EBOV GP) was generated as described previously [3,59]. Virus titers in EBOV ADE cell line were determined as infectious units (IUs) by counting GFP-positive cells. All infectious work with EBOV was performed in the biosafety level 4 laboratory at the Integrated Research Facility of the Rocky Mountain Laboratories, Division of Intramural Research, National Institute of Allergy and Infectious Diseases, National Institutes of Health, Hamilton, Montana, USA.

### Generation of FcγRIIa- or FcγRIIaΔCT-expressing Jurkat T cells

The FcγRIIa gene was PCR-amplified from a full-length cDNA library prepared from K562 cells using the primers, EcoRI-FcγRIIa (5’-GGGAATTCGGATGACTATGGAGACCCAA-3’) and FcγRIIa-XhoI (5’-ATTTCTCGAGTTTGTCATCCACTCAGCAAG-3’). Mutant FcγRIIa lacking its cytoplasmic tail (amino acid positions 241–317) was generated using a PrimeSTAR Mutagenesis Basal Kit (Takara). After sequence confirmation, these PCR products were cloned into a murine leukemia virus-based retroviral vector, pMXs-Puro Retroviral Vector (Cell Biolabs). To generate the retrovirus, 293T-derived Platinum-GP (Plat-GP) cells (Cell Biolabs) were cotransfected with pMXs-puro encoding FcγRIIa or FcγRIIaΔCT and the expression plasmid pCAGGS encoding the VSV G protein using Lipofectamine 2000 (Invitrogen). Forty-eight h later, the culture supernatants containing retroviruses were collected, clarified through 0.45-μm filters, and then used to infect Jurkat T cells. Jurkat T cell lines stably expressing FcγRIIa or FcγRIIaΔCT were selected with RPMI medium containing 10% FCS, 100 U/ml penicillin, 0.1 mg/ml streptomycin, and 10 μg/ml puromycin (Sigma-Aldrich). For some experiments, each Jurkat T cell line was cloned by limiting dilution to enrich the population of FcγRIIa-expressing cells. To check the expression levels of FcγRIIa and FcγRIIaΔCT, these cells were incubated with a mouse anti-CD32 monoclonal antibody (GeneTex) for 1 h at room temperature. After washing 3 times with phosphate-buffered saline (PBS), binding of the primary antibody was detected with Alexa647-conjugated F(ab')2-goat anti-mouse IgG (H+L) (Jackson ImmunoResearch). After further washing 3 times with PBS, the fluorescent intensity of the cells was analyzed using a FACS Canto flow cytometer (BD Biosciences) and FlowJo software (Tree Star).

### ADE assays

EBOV was appropriately diluted to provide 50–100 IUs/50 μl in K562 cells and then incubated for 30 min-1 h at 37°C with or without 10 μg/ml MAbs. The anti-EBOV GP MAb ZGP12/1.1 (IgG2a), which is known to enhance EBOV infection *in vitro*, was used as the ADE MAb [12]. S139/1 (IgG2a), a MAb specific to influenza A virus hemagglutinin, was used as the CTR IgG [60]. K562 and Jurkat T cells were inoculated with EBOV alone or EBOV/MAb mixtures and incubated for 72 h. Virus infectivity was measured by counting the number of GFP-positive cells in FACS and analyzed using FlowJo software. VSV-EBOV GP appropriately diluted to yield 50–100 IUs/50 μl in K562 cells was incubated for 1 h at room temperature with or without 1 μg/ml MAbs, and then inoculated into K562 and Jurkat T cells. Twenty-four h later, GFP-positive cells were counted using an IN Cell Analyzer 2000 (GE Healthcare). To reduce the background (i.e., residual) infectivity of the parent VSV, VSV-EBOV GP was treated with a neutralizing MAb to VSV G protein (VSV-G[N]1–9) before use.

### Purification and DiI-labeling of VLPs

For purification of VLPs, HEK293T cells were transfected with equal amounts of the expression plasmids encoding EBOV or SUDV GP, VP40, and NP using TransIT LT-1 (Mirus) according to the manufacturer's instructions. Forty-eight h after transfection, the culture supernatant was harvested and centrifuged at 3,500 rpm for 15 min at 4°C to remove cell debris. VLPs were precipitated through a 25% sucrose cushion by centrifugation at 11,000 rpm for 1 h at 4°C with an SW32Ti rotor (Beckman). Pelleted VLPs were suspended in PBS, and fractionated through a 20–50% sucrose gradient in PBS at 28,000 rpm with an SW41 rotor (Beckman) for 2 h at 4°C. One ml of 1 μg/ml fractionated VLPs was incubated with 0.6 μl of 100 μM 1,1'-dioctadecyl-3,3,3',3'-tetramethylindocarbocyanine perchlorate (DiI) (Molecular Probes) in the dark for 1 h at room temperature with gentle agitation [6,61].

### Imaging of attachment and internalization of DiI-labeled VLPs

The eGFP-Rab7 fusion protein gene was cloned into a Moloney murine leukemia virus-based retrovirus plasmid [6,62], and recombinant retroviruses for the expression of eGFP-Rab7 were produced and used to infect K562 cells as described above. K562 and Jurkat T cell lines were cultured in 35 mm glass-bottom dishes (MatTek Corporation) precoated with borate buffer containing 0.1 mg/ml poly-L-lysine (Sigma). The cells were washed with 200 μl phenol red-free RPMI (Gibco) and inoculated with 100 μl of 1 μg/ml DiI-labeled VLPs treated with 20 μg/ml ZGP12/1.1 or CTR IgG for 1 h at room temperature, followed by incubation for 30 min on ice. They were then washed twice with the same medium to remove unbound DiI-labeled VLPs and incubated with 200 μl phenol red-free RPMI containing 2% FCS and 4% bovine serum albumin (BSA) for 0 and 2 h at 37°C to analyze DiI-labeled VLP attachment and internalization, respectively. To count the number of DiI-labeled VLPs, the cells were fixed with 4% paraformaldehyde for 15 min at room temperature. Then the nuclei were stained with 1 μg/ml 4',6-diamidino-2-phenylindole, dihydrochloride (DAPI) (Molecular Probes) for 10 min at room temperature. Microscopic images were acquired with a 63× oil objective lens on a Zeiss LSM780 inverted microscope and ZEN 2010 software (Carl Zeiss). For measurement of the number of DiI-labeled VLPs, images of 4–20 optical sections were acquired in 1 micron steps. The number of DiI-labeled VLPs was determined in approximately 100 individual cells using MetaMorph software (Molecular Devices) and the average number per cell was calculated for each condition. For colocalization analysis, the percentage of DiI-labeled VLPs that colocalized with eGFP-Rab7 (Both DiI- and eGFP-positive pixels/DiI-positive pixels × 100) was measured in approximately 100 individual cells using the Coloc module in ZEN 2010 software (Carl Zeiss).

### Dextran uptake assays

One μg/ml DiI-labeled VLPs were treated with 20 μg/ml CTR IgG or ZGP12/1.1 for 1 h at room temperature. K562 and Jurkat T cell lines were cultured in poly-L-lysine-coated glass-bottom culture dishes and incubated with 100 μl untreated, CTR IgG-, or ZGP12/1.1-treated DiI-labeled VLPs for 30 min on ice. The cells were washed twice with 200 μl phenol red-free RPMI and then incubated with 200 μl phenol red-free RPMI containing 2% FCS, 4% BSA, and 0.5 mg/ml Dextran, Alexa Fluor 647, 10,000 MW (Alexa647-labeled Dx10) (Molecular Probes) for 1–2 h at 37°C. After washing twice with 200 μl phenol red-free RPMI to remove surface-unbound DiI-labeled VLPs and Alexa647-labeled Dx10, and the cells were fixed with 4% paraformaldehyde for 15 min at room temperature. Then, the nuclei were stained with 1 μg/ml DAPI for 10 min at room temperature. Internalized DiI-labeled VLPs and Alexa647-labeled Dx10 were analyzed by confocal laser scanning microscopy as described above. The percentage of DiI-labeled VLPs that colocalized with Alexa647-labeled Dx10 (Both DiI- and Alexa647-positive pixels/DiI-positive pixels × 100) was measured in approximately 100 individual cells using the Coloc module in ZEN 2010 software. The number and size of Dx10-positive vesicles were analyzed with MetaMorph software.

### Inhibitor treatments

For infection assays, the Syk inhibitor R788 (Santa Cruz), Src family PTK inhibitor PP2 (Tocris), BTK inhibitor LFM-A13 (Focus Biomolecules), PI3K inhibitor LY294002 (Wako), and Ras inhibitor Manumycin A (Santa Cruz) were used for treatments of K562 cells. R788, LFM-A13, and LY294002 were used at 0.15–40 μM. PP2 and Manumycin A were used at 0.15–10 μM. For imaging analysis, K562 cell lines were cultured in 35 mm glass-bottom dishes precoated with poly-L-lysine, and then treated with 20 μM PP2 for 1 h at 37°C. PP2-treated cells were washed with phenol red-free RPMI and inoculated with untreated, CTR IgG-, or ZGP12/1.1-treated DiI-labeled VLPs for 30 min on ice in the presence of 20 μM PP2 in the same medium. The cells were then washed twice with the same medium and incubated with phenol red-free RPMI containing 2% FCS, 4% BSA, and 20 μM PP2 for 0 and 2 h at 37°C. Then they were fixed and analyzed by confocal laser scanning microscopy as described above. Dimethyl sulfoxide (DMSO, Sigma-Aldrich) or ethanol (Kanto Chemical) was used as a solvent control.

### Generation of Src or Syk knockdown K562 cells

Plat-GP cells (Cell Biolabs) were cotransfected with pRS (retroviral plasmids) encoding human Src or Syk shRNA (ORIGENE) and the expression plasmid pCAGGS encoding the VSV G protein using Lipofectamine 2000 (Invitrogen). ShSrc target sequences were: shSrc1:5’-GGAGGCTTCAACTCCTCGGACACCGTCAC-3’, shSrc2: 5’-AAGAAAGGCGAGCGGCTCCAGATTGTCAA-3’, shSrc3: 5’-GCAGTTGTATGCTGTGGTTTCAGAGGAGC-3’, shSrc4: 5’-CTGGAGGCAATCAAGCAGACATAGAAGAG-3’. ShSyk target sequences were: shSyk1:5’- GAATATGTGAAGCAGACATGGAACCTGCA-3’, shSyk2: 5’- GGAGGAGGCAGAAGATTACCTGGTCCAGG-3’, shSyk3: 5’- TGTCATTCAATCCGTATGAGCCAGAACTT-3’, shSyk4: 5’- CTCTGGCAGCTAGTCGAGCATTATTCTTA-3’. After incubation for 48 h, culture supernatants containing the retroviruses expressing human Src or Syk shRNAs were collected, clarified through 0.45-μm filters, and then used to infect K562 cells. Transduced K562 cell lines were selected with RPMI medium containing 10% FCS, 100 U/ml penicillin, 0.1 mg/ml streptomycin, and 5 μg/ml puromycin (Sigma-Aldrich). To check the knockdown efficiency for Src and Syk, cells were collected and washed once with PBS and treated with lysis buffer (0.1% Nonidet P-40, 150 mM NaCl, 1 mM EDTA, 10 mM Tris HCl, pH 7.8) in the presence of a protease inhibitor cocktail, Complete mini (Roche). Then the lysates were mixed with SDS-PAGE sample buffer (Bio-Rad) with 5% 2-mercaptoethanol (Wako) and boiled for 5 min. The samples were electrophoresed by SDS-PAGE on 5 to 20% gradient polyacrylamide gel, SuperSep Ace (Wako), and separated proteins were blotted on a polyvinylidene difluoride membrane (Millipore). The membrane was blocked for at least 1 h at room temperature with Tris-buffered saline containing 0.1% Tween 20 (TBST) and 1% BSA. Then the membrane was incubated with a rabbit anti-Src antibody (36D10: Cell Signaling) or mouse anti-Syk antibody (4D10.1: Abcam) in TBST containing 1% BSA, followed by incubation with peroxidase-conjugated donkey anti-rabbit IgG (H+L) or peroxidase-conjugated goat anti-mouse IgG (H+L) (Jackson ImmunoResearch), respectively, and visualization by Immobilon Western (Millipore). Band intensities were analyzed with a VersaDoc Imaging System (Bio-Rad) and quantified with Image Lab version 3.0 software (Bio-Rad).

### Phosphorylation assay

One μg/ml purified VLPs were treated with 20 μg/ml ZGP12/1.1 or CTR IgG for 1 h at room temperature. K562 cells were incubated with DMSO or 20 μM PP2 for 1 h at 37°C. Untreated or PP2-treated K562 cells were inoculated with untreated, CTR IgG-, or ZGP12/1.1-treated VLPs and incubated for 0, 10, 30, or 60 min at 37°C. At each time point, cells were collected and washed once in PBS and treated with lysis buffer (0.1% Nonidet P-40, 150 mM NaCl, 1 mM EDTA, 10 mM Tris HCl, pH 7.8) in the presence of a protease inhibitor cocktail, Complete mini (Roche), and a phosphatase inhibitor cocktail, PhosSTOP (Roche). Then the lysates were mixed with SDS-PAGE sample buffer (Bio-Rad) with 5% 2-mercaptoethanol (Wako) and boiled for 5 min. The samples were electrophoresed by SDS-PAGE on 5 to 20% gradient polyacrylamide gel, SuperSep Ace (Wako), and separated proteins were blotted on a polyvinylidene difluoride membrane (Millipore). The membrane was blocked for at least 1 h at room temperature with Tris-buffered saline containing 0.1% Tween 20 (TBST) and 1% BSA. Then the membrane was incubated with a phospho-Src family (Tyr416) antibody (Cell Signaling) in TBST containing 1% BSA, followed by visualization using peroxidase-conjugated donkey anti-rabbit IgG (H+L) (Jackson ImmunoResearch) and Immobilon Western (Millipore). Band intensities were analyzed with a VersaDoc Imaging System (Bio-Rad) and quantified with Image Lab version 3.0 software (Bio-Rad).

### Statistical analysis

All data were analyzed using Excel software. In all experiments, Student’s *t*-test was used to evaluate statistical differences. *P* values of less than 0.05 were considered to be significant.

## Supporting Information

S1 Fig EBOV ADE in K562 cells. K562 cells were infected with VSV-EBOV GP or EBOV following incubation with CTR IgG or ZGP12/1.1 for 30 min-1 h at 37°C. After incubation for 24 (VSV-EBOV GP) or 72 (EBOV) h, GFP-positive cells were counted and IUs of viruses were determined (A). The relative percentage of infectivity was calculated by setting the IU value of the viruses in CTR IgG-treated cells to 100% (B). The mean and standard deviation of three independent experiments are shown.
(PDF)

S2 Fig Effects of PP2 on the VSV-EBOV GP infectivity in K562 cell lines stably expressing C-type lectin. K562/DC-SIGN, K562/hMGL, and mock control K562 cells were treated with DMSO or PP2 for 1 h at 37°C and infected with VSV-EBOV GP in the presence of the inhibitor. After incubation for 24 h, GFP-positive cells were counted. The relative percentage of infectivity was calculated by setting the IU value of the virus in DMSO-treated cells to 100%. The mean and standard deviation of three independent experiments are shown.
(PDF)

S3 Fig Magnified images of DiI-labeled VLPs and eGFP-Rab7 shown in Fig 6. K562 cells expressing eGFP-Rab7 were incubated with DMSO or PP2 for 1 h at 37°C. Untreated (Control), CTR IgG-, and ZGP12/1.1-treated DiI-labeled VLPs were inoculated into the cells and incubated for 30 min on ice. After adsorption, the cells were incubated for 2 h at 37°C in the presence of DMSO (A) or PP2 (B). VLPs (red) and eGFP-Rab7 (green) in the cytoplasm were monitored by confocal laser scanning microscopy. Scale bars represent 10 μm. Nuclei of cells are visualized with DAPI (blue).
(PDF)

S4 Fig Magnified images of DiI-labeled VLPs and Alexa647-labeled Dx10 shown in Fig 7. K562 cells were incubated with DMSO (A) or PP2 (B) for 1 h at 37°C. Untreated (Control), CTR IgG-, and ZGP12/1.1-treated DiI-labeled VLPs were inoculated into cells and incubated for 30 min on ice. After adsorption, cells were incubated with Alexa647-labeled Dx10 for 1 h at 37°C in the presence of DMSO (A) or PP2 (B). VLPs (red) and Dx10 (green) in the cytoplasm were monitored by confocal laser scanning microscopy. Scale bars represent 10 μm. Nuclei of cells are visualized with DAPI (blue).
(PDF)

S5 Fig Attachment, uptake, and localization of DiI-labeled SUDV VLPs. Untreated (Control), CTR IgG-, and ZGP12/1.1-treated DiI-labeled SUDV VLPs were inoculated into K562 cell lines and SUDV VLPs (red) on the cell surface at 0 h (A, D) and VLPs (red) and eGFP-Rab7 (B, E) (green) or Dx10 (C, F) (green) in the cytoplasm at 2 h after adsorption were monitored by confocal laser scanning microscopy. Scale bars represent 10 μm. Nuclei of cells are visualized with DAPI (blue). The number of SUDV VLPs on the cell surface (D) and the colocalization of SUDV VLPs (DiI) and eGFP-Rab7 (E) or Dx10 (F) signals were quantified. The mean and standard deviation of three independent experiments are shown. Statistical analysis was performed using Student’s *t*-test (*p<0.05).
(PDF)
