# Supplementary figures and images for: Fcγ-receptor IIa-mediated Src Signaling Pathway Is Essential for the Antibody-Dependent Enhancement of Ebola Virus Infection
Source: PLoS Pathog. 2016 Dec 30;12(12):e1006139. doi: 10.1371/journal.ppat.1006139 (PMC5231391; doi:10.1371/journal.ppat.1006139)

**A**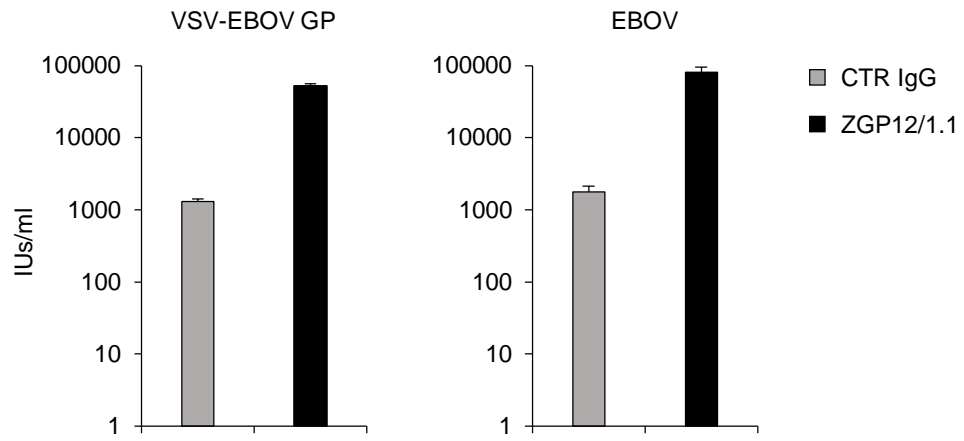**B**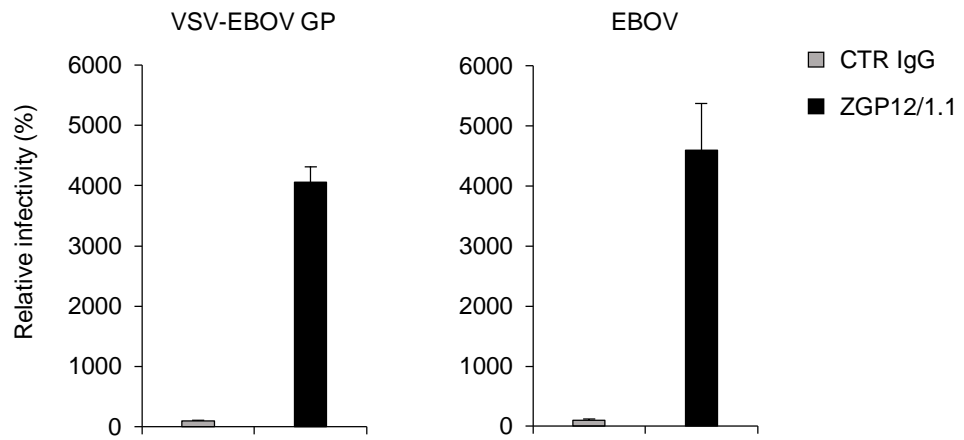

Supplement: S1 Fig — K562 cells were infected with VSV-EBOV GP or EBOV following incubation with CTR IgG or ZGP12/1.1 for 30 min-1 h at 37°C. After incubation for 24 (VSV-EBOV GP) or 72 (EBOV) h, GFP-positive cells were counted and IUs of viruses were determined (A). The relative percentage of infectivity was calculated by setting the IU value of the viruses in CTR IgG-treated cells to 100% (B). The mean and standard deviation of three independent experiments are shown. (PDF) [file ppat.1006139.s001.pdf]

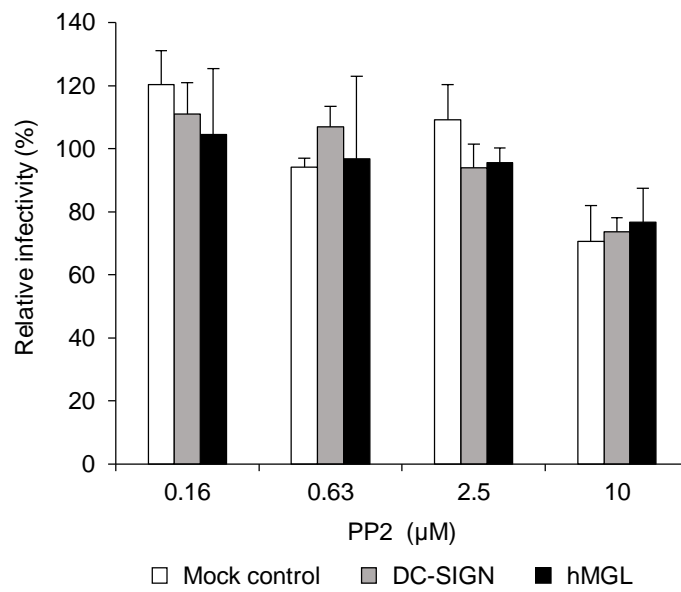

Supplement: S2 Fig — K562/DC-SIGN, K562/hMGL, and mock control K562 cells were treated with DMSO or PP2 for 1 h at 37°C and infected with VSV-EBOV GP in the presence of the inhibitor. After incubation for 24 h, GFP-positive cells were counted. The relative percentage of infectivity was calculated by setting the IU value of the virus in DMSO-treated cells to 100%. The mean and standard deviation of three independent experiments are shown. (PDF) [file ppat.1006139.s002.pdf]

A

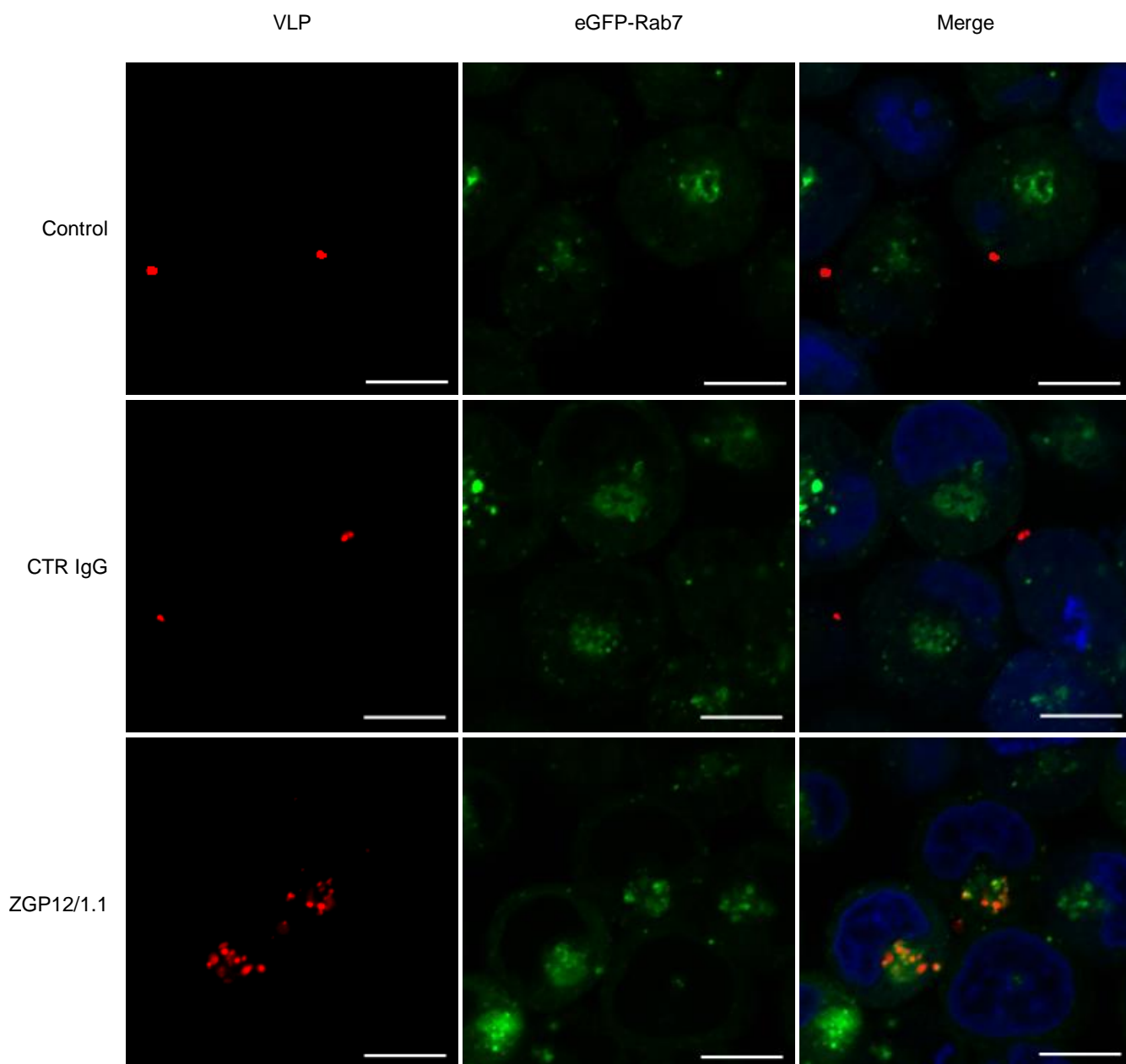

B

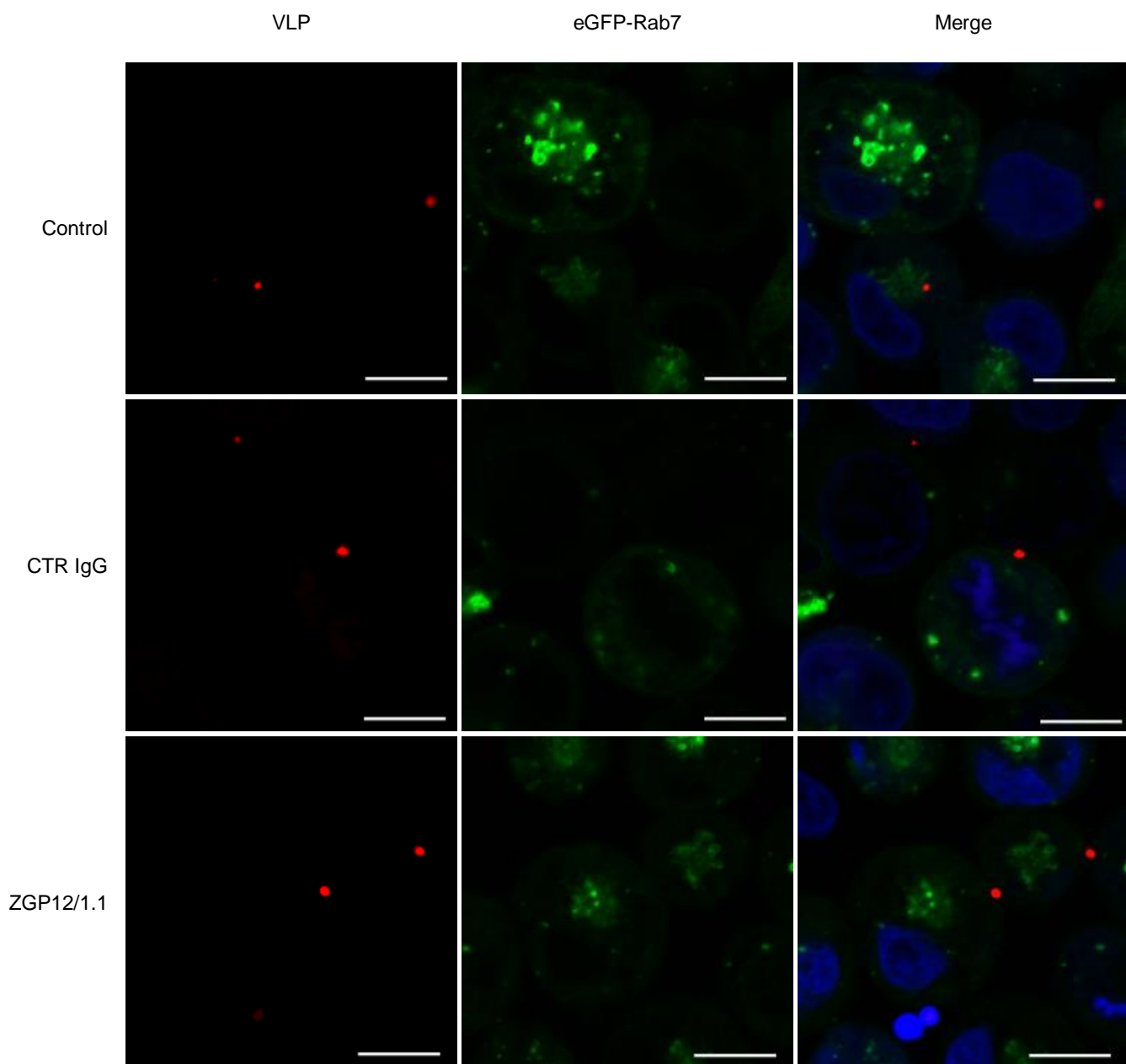

Supplement: S3 Fig — K562 cells expressing eGFP-Rab7 were incubated with DMSO or PP2 for 1 h at 37°C. Untreated (Control), CTR IgG-, and ZGP12/1.1-treated DiI-labeled VLPs were inoculated into the cells and incubated for 30 min on ice. After adsorption, the cells were incubated for 2 h at 37°C in the presence of DMSO (A) or PP2 (B). VLPs (red) and eGFP-Rab7 (green) in the cytoplasm were monitored by confocal laser scanning microscopy. Scale bars represent 10 μm. Nuclei of cells are visualized with DAPI (blue). (PDF) [file ppat.1006139.s003.pdf]

A

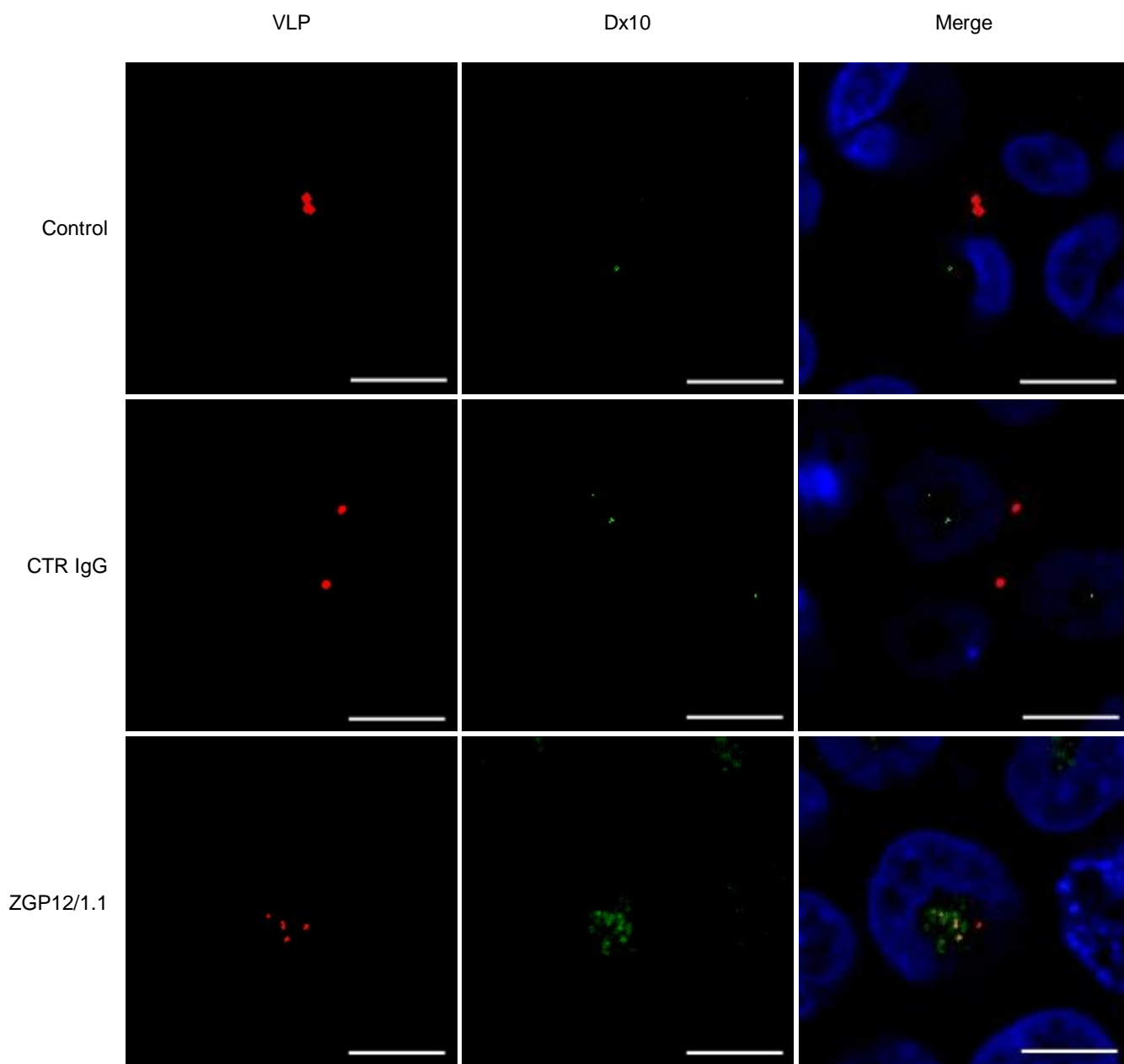

B

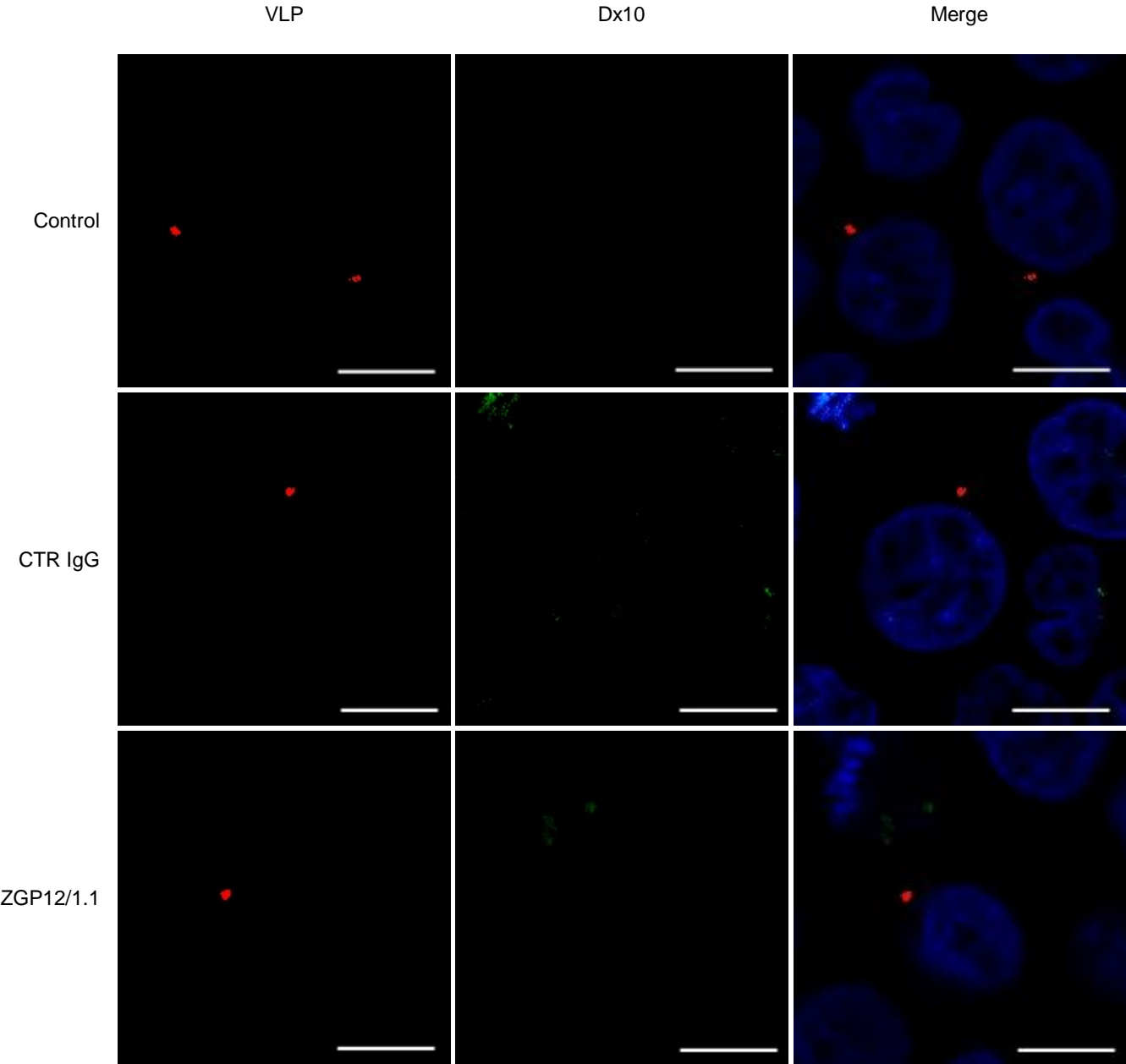

Supplement: S4 Fig — K562 cells were incubated with DMSO (A) or PP2 (B) for 1 h at 37°C. Untreated (Control), CTR IgG-, and ZGP12/1.1-treated DiI-labeled VLPs were inoculated into cells and incubated for 30 min on ice. After adsorption, cells were incubated with Alexa647-labeled Dx10 for 1 h at 37°C in the presence of DMSO (A) or PP2 (B). VLPs (red) and Dx10 (green) in the cytoplasm were monitored by confocal laser scanning microscopy. Scale bars represent 10 μm. Nuclei of cells are visualized with DAPI (blue). (PDF) [file ppat.1006139.s004.pdf]

A

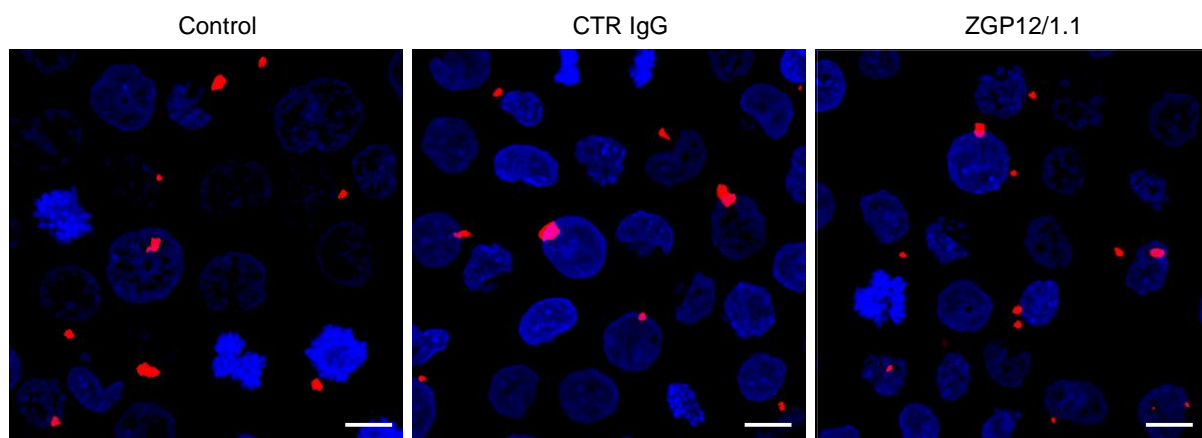

B

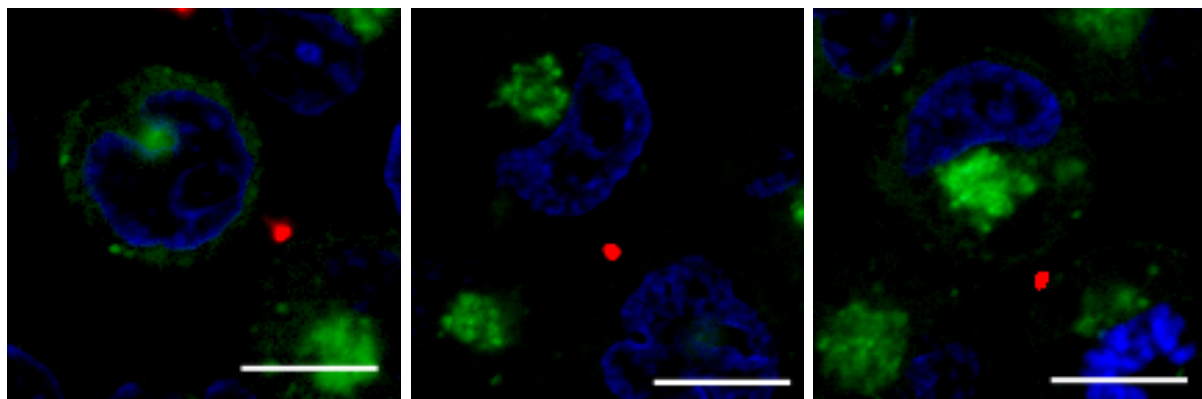

C

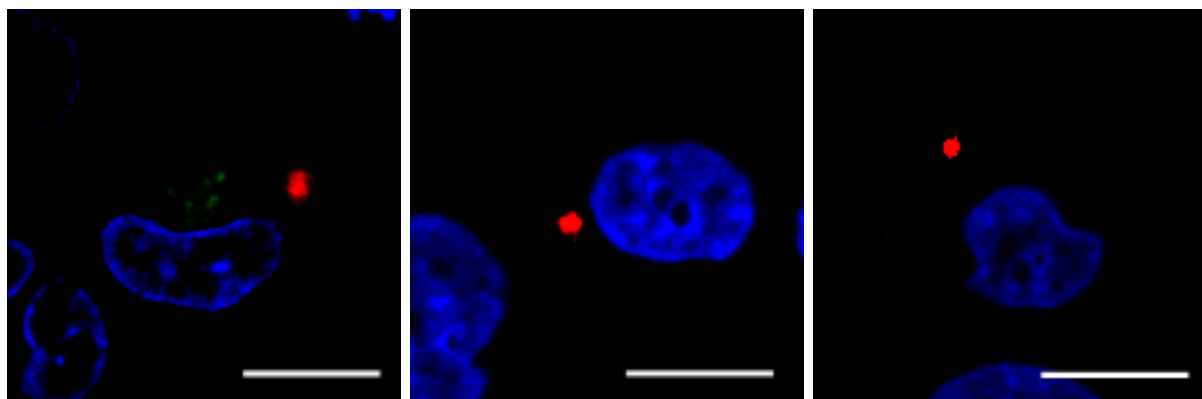

D

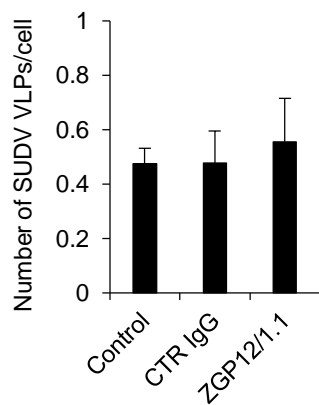

E

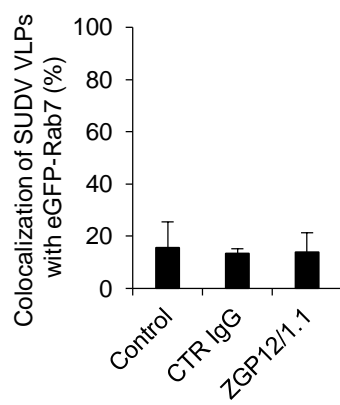

F

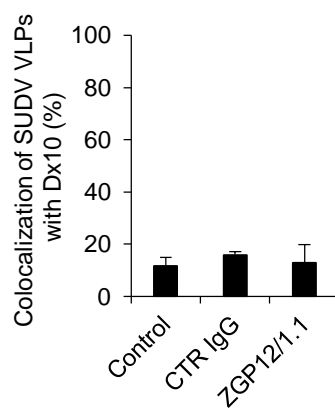

Supplement: S5 Fig — Untreated (Control), CTR IgG-, and ZGP12/1.1-treated DiI-labeled SUDV VLPs were inoculated into K562 cell lines and SUDV VLPs (red) on the cell surface at 0 h (A, D) and VLPs (red) and eGFP-Rab7 (B, E) (green) or Dx10 (C, F) (green) in the cytoplasm at 2 h after adsorption were monitored by confocal laser scanning microscopy. Scale bars represent 10 μm. Nuclei of cells are visualized with DAPI (blue). The number of SUDV VLPs on the cell surface (D) and the colocalization of SUDV VLPs (DiI) and eGFP-Rab7 (E) or Dx10 (F) signals were quantified. The mean and standard deviation of three independent experiments are shown. Statistical analysis was performed using Student’s t-test (*p<0.05). (PDF) [file ppat.1006139.s005.pdf]
